# Development of Porous and Flexible PTMC Membranes for In Vitro Organ Models Fabricated by Evaporation-Induced Phase Separation

**DOI:** 10.3390/membranes10110330

**Published:** 2020-11-05

**Authors:** Thijs Pasman, Danielle Baptista, Sander van Riet, Roman K. Truckenmüller, Pieter S. Hiemstra, Robbert J. Rottier, Dimitrios Stamatialis, André A. Poot

**Affiliations:** 1 Department of Biomaterials Science and Technology, Technical Medical (TechMed) Centre, Faculty of Science and Technology, University of Twente, 7522 NB Enschede, The Netherlands; t.pasman@utwente.nl (T.P.); d.stamatialis@utwente.nl (D.S.); 2 Department of Instructive Biomaterials Engineering, MERLN Institute for Technology-Inspired Regenerative Medicine, Maastricht University, 6229 ER Maastricht, The Netherlands; danielle.baptista@maastrichtuniversity.nl (D.B.); r.truckenmuller@maastrichtuniversity.nl (R.K.T.); 3 Department of Pulmonology, Leiden University Medical Centre, 2300 RC Leiden, The Netherlands; s.van_riet@lumc.nl (S.v.R.); p.s.hiemstra@lumc.nl (P.S.H.); 4 Department of Pediatric Surgery, Erasmus MC-Sophia Children’s Hospital, 3000 CB Rotterdam, The Netherlands; r.rottier@erasmusmc.nl

**Keywords:** membranes, poly(trimethylene carbonate) (PTMC), evaporation-induced phase separation (EIPS), photo-crosslinking, in vitro organ models

## Abstract

Polymeric membranes are widely applied in biomedical applications, including in vitro organ models. In such models, they are mostly used as supports on which cells are cultured to create functional tissue units of the desired organ. To this end, the membrane properties, e.g., morphology and porosity, should match the tissue properties. Organ models of dynamic (barrier) tissues, e.g., lung, require flexible, elastic and porous membranes. Thus, membranes based on poly (dimethyl siloxane) (PDMS) are often applied, which are flexible and elastic. However, PDMS has low cell adhesive properties and displays small molecule ad- and absorption. Furthermore, the introduction of porosity in these membranes requires elaborate methods. In this work, we aim to develop porous membranes for organ models based on poly(trimethylene carbonate) (PTMC): a flexible polymer with good cell adhesive properties which has been used for tissue engineering scaffolds, but not in in vitro organ models. For developing these membranes, we applied evaporation-induced phase separation (EIPS), a new method in this field based on solvent evaporation initiating phase separation, followed by membrane photo-crosslinking. We optimised various processing variables for obtaining form-stable PTMC membranes with average pore sizes between 5 to 8 µm and water permeance in the microfiltration range (17,000–41,000 L/m^2^/h/bar). Importantly, the membranes are flexible and are suitable for implementation in in vitro organ models.

## 1. Introduction

With increasing costs to develop new medicines and treatments, and ethical and scientific concerns related to the use of animal models, there is an increasing need for developing in vitro organ-mimicking models [1]. These models should generate more reliable results than traditional models, such as those based on cell cultures in dishes on tissue culture plastic and those in Transwell^®^ inserts under static conditions, since they would consist of functional tissue units that are actively perfused, which better represent the native tissue.

Polymeric membranes are a vital part of many organ models [1,2,3,4,5,6,7,8,9,10]. They provide a surface for the cells to attach and grow and often (partly) substitute the extracellular matrix (e.g., the basal membrane). They often require specific coating for the growth of (primary) cells. Besides, the membrane properties should promote physiological cell behaviour, and thus match the properties of the desired tissue or organ. Importantly, the membranes need to be porous to allow communication between the cells of co-culture models and enable nutrient, metabolite and gas transport to and from the cells. Moreover, the membranes used in organ models of dynamic tissues, such as the lung and heart, need to match the mechanical properties of these organs since these properties affect cellular behaviour [1].

Many of the current in vitro systems include commercial membranes based on poly (ethylene terephthalate) (PET) or poly (bisphenol-A-carbonate) (PC) [1]. Most of these membranes are made porous by ion track etching, which provides great and independent control over pore size and density, and enables a wide range of membrane pore sizes (i.e., 0.4–8 µm) [1]. PET and PC are transparent, which is beneficial for cell imaging techniques. However, both have relatively high elastic moduli (E-moduli), of 2–3 GPa [11] and 2–2.4 GPa [12], respectively. These moduli are quite appropriate for matching those of cortical bone (6–34 GPa) [13,14,15,16,17], cancellous bone (0.1 to 2 GPa) [15,16], and specific ligaments (366 MPa) [16], but far exceed the moduli of most tissues which are in the range of several kPa to MPa [16,17,18,19].

Other culture systems implement membranes based on poly (dimethyl siloxane) (PDMS) [1,2,3,4,6,20,21]. The E-modulus of PDMS is easily tunable from 4 kPa to several MPa [22], which facilitates cyclic stretch of the membranes and cells [2,4,6]. Moreover, PDMS is transparent and easy to prepare [23]. However, the fabrication of porous PDMS membranes suitable for cell culturing is difficult [24,25], requiring rather elaborate methods (i.e., mostly soft lithography) [1]. Furthermore, their pore density is often low [2,10], which potentially prevents optimal cell communication and nutrient delivery to cells. Besides, cell attachment on PDMS is low [26]. Therefore, PDMS needs additional coatings, surface modifications, or both, to accommodate cell growth [26]. Finally, PDMS is known for its ad- and absorption of small molecules, potentially affecting outcomes of (drug) studies [27,28].

In this work, we describe the development of membranes for organ-on-chip (OoC) models based on the polymer poly(trimethylene carbonate) (PTMC), which is transparent [23], non-cytotoxic [29,30] and has good cell adhesive properties [30,31,32]. PTMC adsorbs cell culture medium components [31], which is beneficial for cell adhesion but it can also be coated [33,34,35] for improving cell adhesion. This could be useful for (primary) cells that require specific coatings. Earlier studies have also shown that PTMC-based structures (e.g., scaffolds and films) have good mechanical properties, such as high elongation at break, good toughness, relatively low E-moduli compared to PC and PET and low permanent deformation [29,32,33]. Moreover, the mechanical properties of crosslinked PTMC networks can be tuned to match those of the targeted tissue by changing the molecular weight of the PTMC [36] and the crosslinking conditions [29,32]. In addition, their mechanical properties are good and remain unchanged when wetted [33,37,38] due to a low water uptake (i.e., <2%) [32,37,39]. Furthermore, it is biodegradable via surface erosion, while retaining its properties until degradation is almost complete [32,39,40,41], which is beneficial for in vivo applications [34,41,42]. Furthermore, its degradation does not produce any toxic products [29,40]. All these characteristics have made it an interesting and successful material for scaffolds, films and porous membranes for biomedical applications, both in vitro [30,31,32] and in vivo [34,41,42]. Nevertheless, PTMC is amorphous and has a low glass transition temperature (Tg) of −17 °C [31,40]. Therefore, crosslinking of the PTMC is needed to preserve its porous structure. Earlier studies implemented temperature-induced phase separation (TIPS) [43] for the fabrication of porous PTMC membranes and liquid-induced phase separation (LIPS) [31] for porous PTMC scaffolds. However, the fabrication of membranes by TIPS was not reproducible [43]. LIPS resulted in reproducible PTMC scaffolds for cell culture, which could be crosslinked by gamma-irradiation. Nevertheless, the process duration of crosslinking was rather long leading to a substantial loss of membrane porosity [31]. Photo-crosslinking of PTMC scaffolds is possible by using crosslinking agents [32,39]. However, crosslinking the LIPS-based PTMC scaffolds would not be straightforward due to the large volumes of non-solvents used with LIPS, which could lead to solvation and loss of polymer dope components (i.e., the crosslinking agents).

**Scheme 1:**
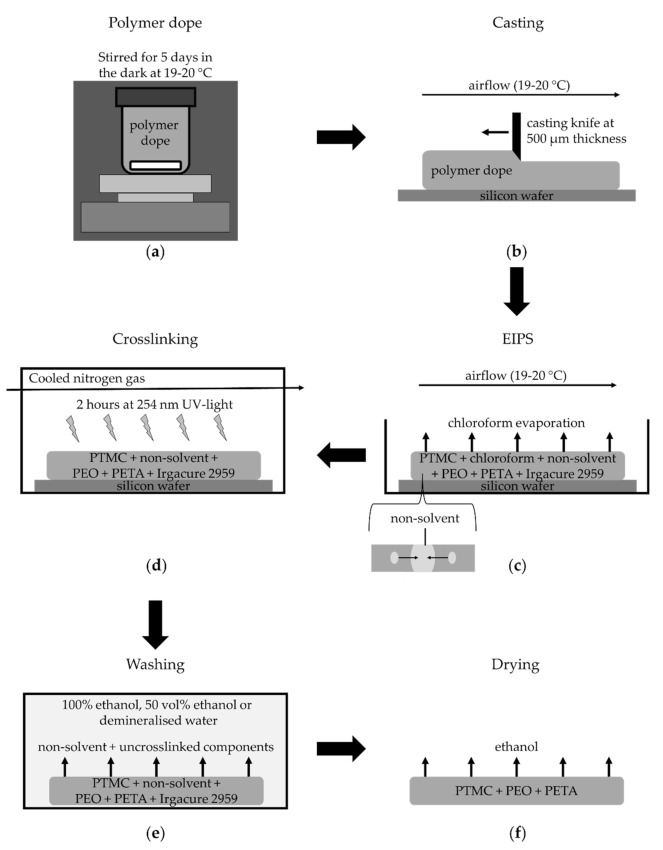
Schematic of PTMC membrane fabrication by EIPS. (**a**) The polymer dope is stirred for five days at 19–20 °C in the dark. (**b**) The dope is then cast on silicon wafers in a 500 µm thick layer using a casting knife. (**c**) The cast polymer layers are kept in a box for 90 min where chloroform evaporation and EIPS occurs. (**d**) The membranes are then crosslinked in a UV-box for two hours at 254 nm UV-light with cooled nitrogen gas flowing through the UV-box. (**e**) Membranes are then washed to remove the non-solvent and any other uncrosslinked components. (**f**) Finally, the membranes are rinsed with ethanol, dried and stored.

In this study, we aim to overcome the above limitations by preparing crosslinked PTMC membranes via evaporation-induced phase separation (EIPS) combined with photo-crosslinking (Scheme 1). For EIPS, a polymer dope which contains a volatile solvent and a non-solvent for the polymer is cast on a suitable surface. Evaporation of the volatile solvent increases the concentration of polymer and non-solvent to a point where phase separation occurs. The non-solvent, which is non-volatile or significantly less volatile than the solvent, coalesces into droplets inside the polymer layer and is present long enough for solidification of the polymer. In this study, polymer dopes containing PTMC and poly (ethylene oxide) (PEO), the latter as pore former and pore stabiliser [31,37], chloroform (solvent) and a non-solvent (i.e., ethanol, propanol, butanol or hexanol), are cast on silicon wafers. Immediately after EIPS, the membranes can be photo-crosslinked with UV-light to preserve the porosity of the amorphous PTMC structures. To enable photo-crosslinking, the polymer dope also contains pentaerythritol triacrylate (PETA) and Irgacure 2959 as crosslinking agents, as they have been used to successfully photo-crosslink porous and cell-adhesive PTMC-based scaffolds [32,39]. After crosslinking, the solidified polymer layer is washed to remove the non-solvent, which reveals the pores created by the non-solvent. Volumes of non-solvent used in EIPS are much smaller than the non-solvent bath used in LIPS. Thus, we anticipate that, with EIPS, it is less likely that components such as the crosslinking agents dissolve into the non-solvent.

Here, we tailored the PTMC membrane morphology and porosity by a detailed investigation of various EIPS parameters, including the environmental conditions (e.g., humidity and temperature), the type of non-solvent (a series of alcohols) and the polymer dope composition (the molecular weight (MW) of PTMC and the amount of non-solvent). The developed membranes were characterised in detail concerning the network chemistry, their mechanical properties, morphology (i.e., roughness, porosity, pore size) and transport properties (water transport). Besides, they were compared to membranes used in commercial inserts (PET and PC) and to PDMS membranes reported in literature.

## 2. Materials and Methods

### 2.1. Materials

Trimethylene carbonate (TMC, Boehringer Ingelheim, Ingelheim, Germany), stannous octoate (Sn(Oct)_2_, Spectrum Chemical MFG Corp., New Brunswick, NJ, USA), chloroform (Merck Millipore, Darmstadt, Germany, Emparta cat. 1.07024.2500, analytical grade), ethanol for membrane washing (Boom B.V., Meppel, The Netherlands, cat. 84050065.5000), ethanol for PTMC precipitation and as non-solvent (Merck Millipore, Darmstadt, Germany, Emsure cat. 1.00983.1000, analytical grade), propan-1-ol (Sigma-Aldrich, Darmstadt, Germany, cat. 279544), butan-1-ol (Sigma-Aldrich, Darmstadt, Germany, cat. 281549), hexan-1-ol (Sigma-Aldrich, Darmstadt, Germany, cat. 471402), poly(ethylene oxide) (PEO) 5,000,000 g/mol (Sigma-Aldrich, Darmstadt, Germany, cat. 189472), pentaerythritol triacrylate (PETA) (Sigma-Aldrich, Darmstadt, Germany, cat. 246794), 2-Hydroxy-4′-(2-hydroxyethoxy)-2-methylpropiophenone (Irgacure 2959) (Sigma-Aldrich, Darmstadt, Germany, cat. 410896) and deuterated chloroform (Sigma-Aldrich, Darmstadt, Germany, cat. 151823) were all used as received. Ultrapure water (MilliQ water) was produced by a Millipore Advantage A10 (Merck Millipore, Darmstadt, Germany). Additional information about the function and MW of the components used in the polymer dopes can be found in Table 1.

### 2.2. Poly(Trimethylene Carbonate) (PTMC) Synthesis

Linear PTMC was synthesised by ring-opening polymerisation of TMC for three days at 130 °C under vacuum in a silanised, dry glass ampoule. MilliQ water (weight ratio of TMC to water of 180:1 and 90:1) and Sn(Oct)_2_ (weight ratio of TMC to Sn(Oct)_2_ of 90:1) were added as initiator and catalyst, respectively. Afterwards, the PTMC was dissolved in chloroform and precipitated in a ten-fold volume of cold ethanol for purification. It was then dried under vacuum at ±20 °C until constant weight. 

One-point viscometry was used to determine the weight average molecular weight, i.e., the $M\bar{v}$, of the PTMC. The $M\bar{v}$ of the PTMC is referred to in the following as MW, similar to the MW of the other polymers. Viscometry measurements were performed on PTMC dissolved in chloroform at 25 °C using a 0C Ubbelohde Viscometer (*N* = 3). The intrinsic viscosities were determined by calculating the relative and specific viscosities and using the Solomon-Ciuta equation. We made PTMC with two different intrinsic viscosities, i.e., 8.3 dL/g and 4.6 dL/g. Using the Mark-Houwink equation (see Equation (1) below), with ‘K’ = 2.43 × 10^−4^ and ‘a’ = 0.74 [40], the MW’s of the two PTMC batches were estimated to be 1300 kg/mol and 600 kg/mol, respectively.

(1) $$\mathrm{Intrinsic}\;\mathrm{viscosity}\;\left[\eta \right]{\;=\;K\;\times \;\mathrm{MW}}^{a}$$

### 2.3. Membrane Fabrication

Polymer dopes with different compositions were prepared in glass jars. They always contained 3 wt% of PTMC in chloroform and 0.3 wt% PEO of 5000 kg/mol (Table 1), according to Papenburg et al. [31]. PETA and Irgacure 2959 (0.2 wt% and 0.01 wt% of the total polymer dope, respectively) were added as crosslinking aid [32] and photoinitiator [39], respectively. The remaining part of the dopes comprised of solely chloroform or a combination of a non-solvent (i.e., ethanol, propanol, butanol or hexanol) and chloroform. We made two different types of dopes:

- For the investigation of the effect of non-solvent type on the formation of porous membranes, we used polymer dopes of PTMC of 1300 kg/mol and different alcohols as non-solvents. These dopes always contained 3 wt% non-solvent and 93.5 wt% chloroform.
- For the investigation of the effect of the MW of PTMC and the non-solvent amount on the formation of porous membranes, polymer dopes contained PTMC of either 1300 kg/mol or 600 kg/mol. Furthermore, they contained different amounts of hexanol as a non-solvent, i.e., 0, 3, 6 or 9 wt% of the total polymer dope. Membranes fabricated from PTMC with a MW of 600 kg/mol and 0, 3, 6 or 9 wt% hexanol are in the following referred to as M0, M1, M2 and M3, respectively (Table 2).

Jars containing the polymer dopes were sealed with a lid and parafilm, and the polymer dopes were stirred at an ambient temperature of 19–20 °C in the dark for five days (Scheme 1a). The membranes were cast by pouring the polymer solutions on silicon wafers, after which a casting knife, adjusted to a casting gap of 500 µm, was used to create a 500 µm thick layer (Scheme 1b). Casting and fabrication of the membranes were done in a fume hood at 19–20 °C with a constant airflow of 0.75 m/s. This airflow was applied across the surface of the cast polymer layers to facilitate the removal of chloroform vapours above the membrane.

The different dopes were cast in different conditions:

- Polymer dopes with PTMC of 1300 kg/mol and different alcohols as non-solvents were cast at a humidity of 50%. Multiple membranes were cast (*N* = 3).
- The dopes with different hexanol amounts, including those for M0–M3, were cast at 60% humidity, created by a humidifier (PureMate^®^ PM 702, PureMate, Birmingham, UK). Moreover, the silicon wafers containing cast polymer layers of those dopes were immediately placed on a cold glass plate of −25 °C and 5 mm thick. The cold glass plate was not kept cool and thus warmed up to ambient temperature over time. Casting was performed at least five times for each condition with multiple membranes cast every time. Samples were taken from different membranes cast on different days.

The cast membranes were then left for 90 min in a box (Scheme 1c) in the conditions described above. During this time, EIPS occurred. 

The membranes and silicon wafers were then placed in a UV-box (UltraLum CEX 1500 crosslinking cabinet, Ultra Lum Enterprises, Inc., Claremont, CA, USA) for photo-crosslinking for two hours (UV-light at 254 nm and an intensity of 5 mW/cm^2^, measured using an optical power meter (Newport 1916-C, Irvine, CA, USA) at a distance of 3 cm to the light source) (Scheme 1d). Nitrogen gas, cooled by flowing through a cold trap submerged in liquid nitrogen, was flowing into the UV-machine at a rate of 4 L/min to prevent reaction of the crosslinking agents with oxygen. 

Afterwards, the membranes were washed (Scheme 1e) to remove the non-solvent, uncrosslinked polymer and PETA, and residual Irgacure 2959.

- The membranes prepared with PTMC of 1300 kg/mol and different alcohols were washed in an excess of demineralised water for five days (water refreshed once every day).
- The membranes prepared with different hexanol amounts, including M0–M3, were first put in an excess of 100% ethanol overnight since hexanol does not mix well with water but is miscible with ethanol. After one day, the ethanol was exchanged for 50 vol% ethanol in demineralised water for one day, followed by four days of demineralised water (water replaced once per day). Then, the membranes were again placed in 100% ethanol overnight to remove any residual hexanol and to exchange the water in the membranes with ethanol for better drying.

Finally, all membranes were rinsed with 100% ethanol several times and placed on Teflon paper. They were dried until constant weight at 19–20 °C, vacuum sealed and stored at −25 °C (Scheme 1f).

### 2.4. The Polymer Network in the Membranes

#### 2.4.1. Gel Content

To assess whether crosslinking was successful, we performed gel content measurements. Samples (*N* ≥ 3) were cut from membranes that were washed and dried. The mass of the samples was determined (M_Before_), and samples were then submerged in an excess of chloroform for two days. Afterwards, the samples were dried until constant weight and weighed again (M_After_). The gel content was then determined with Equation (2).

(2) $$\mathrm{Gel}\;\mathrm{content}\;=\;\frac{M_{\mathrm{After}}}{M_{\mathrm{Before}}}\;\times \;100\%$$

#### 2.4.2. Proton Nuclear Magnetic Resonance Spectroscopy (^1^H-NMR)

We performed a qualitative proton nuclear magnetic resonance spectroscopy (^1^H-NMR) analysis of the sol fraction of membranes to determine whether uncrosslinked PTMC, PEO or PETA, as well as residual Irgacure 2959, hexanol or chloroform, were still present in washed membranes. Samples of the membranes (*N* ≥ 5) were cut and placed in deuterated chloroform as a solvent at a concentration of 15 mg/mL. Samples containing 2.5 mg/mL of one of the pure components were used as references (*N* = 3). The samples were then shaken gently for two days in the dark. Afterwards, in the case of the membranes, the membrane samples were taken out of the chloroform. The chloroform (including any present molecules) as well as the reference samples, were then added to NMR tubes (Sigma-Aldrich, Darmstadt, Germany, cat. Z274526-1PAK, Wilmad 527-PP-8) for analysis in a Bruker Ascend 400 (Avance III 400 MHz NMR spectrometer) (Bruker, Billerica, MA, USA).

### 2.5. Mechanical Properties of Membranes

The E-modulus of membranes was determined by placing dry dumbbell-shaped samples (50 mm × 4 mm) in a Zwick/Roell Z020/SND tensile tester (Zwick/Roell, Ulm, Germany) with a 500 N load cell and a grip-to-grip separation of 30 mm. Stress-strain measurements were then done by applying a preload of 0.01 N, followed by elongation of the samples at a speed of 10 mm/min at 19–20 °C. The strain was measured with extensometers. The E-moduli were then determined as the slope of the initial linear phase of the stress-strain curves (*N* ≥ 2 from different membranes).

### 2.6. Porosity, Morphology and Water Transport

#### 2.6.1. Overall Porosity and Thickness

Porosity of the membranes was measured by punching circular samples with a diameter of 26 mm of membranes after washing and drying. Samples from different membranes were used (*N* ≥ 4). Thickness (*N* ≥ 6) of the samples was then measured with a calliper (Model ID-C112B, Mitutoyo Corp., Kawasaki, Japan). Subsequently, the porosity was calculated with Equations (3)–(6):

(3) $$\mathrm{Porosity}\;=\;\frac{\mathrm{total}\;\mathrm{sample}\;\mathrm{volume}\;-\;\mathrm{skeletal}\;\mathrm{volume}}{\mathrm{total}\;\mathrm{sample}\;\mathrm{volume}}\;\times \;100\%$$

The total sample volume and skeletal volume were determined by:

(4) $${\mathrm{Total}\;\mathrm{sample}\;\mathrm{volume}\;=\;\pi \;\times \;r}^{2}\;\mathrm{of}\;\mathrm{sample}\;\times \;\mathrm{thickness}\;\mathrm{of}\;\mathrm{sample}$$

(5) $$\mathrm{Skeletal}\;\mathrm{volume}\;=\;\frac{\mathrm{weight}\;\mathrm{of}\;\mathrm{sample}}{\mathrm{overall}\;\mathrm{density}\;\mathrm{of}\;\mathrm{membrane}}$$

(6) $$\mathrm{Overall}\;\mathrm{density}\;\mathrm{of}\;\mathrm{membrane}\;=\;\left(86.33\%\;\times \;\mathrm{density}\;\mathrm{of}\;\mathrm{PTMC}\right)\;+\;\;\left(8.63\%\;\times \;\mathrm{density}\;\mathrm{of}\;\mathrm{PEO}\right)\;+\;(5.04\%\;\times \;\mathrm{density}\;\mathrm{of}\;\mathrm{PETA})$$

The skeletal volume was determined with Equation (5). For the overall density of the membranes, it was assumed that there was a complete removal of chloroform, Irgacure and hexanol, while all of the initial PTMC, PEO and PETA was still present in the membranes. Thus, the percentages in Equation (6) represent the wt% of PTMC, PEO and PETA, respectively, in the membranes after removal of chloroform, Irgacure and hexanol. Densities of PTMC, PEO and PETA are 1.31 g/mL, 1.21 g/mL and 1.18 g/mL, respectively.

#### 2.6.2. Membrane Morphology

Membrane morphology was assessed by Scanning Electron Microscopy (SEM). Dry samples of the membranes were gold-sputtered under vacuum in a sputter coater (Cressington 108 Auto, Cressington, Watford, UK) equipped with a pure gold target (Aurion, Wageningen, The Netherlands, cat. 91017-AU) at 10 mA for 60 s. Subsequent imaging was performed on a JSM-6010LA (JEOL, Tokyo, Japan) at 5 kV. Pictures of M0-M3 membranes (*N* ≥ 7) taken from membranes cast on different days were analysed with ImageJ for pore analysis. First, the average pore diameter was calculated for each pore, after which these values were used to determine the average pore diameter ± SD.

#### 2.6.3. Water Transport Across the Membranes

Circular samples of membranes with a diameter of 26 mm were punched. PET membranes with 0.4 µm pores (Sigma-Aldrich, Darmstadt, Germany, Corning, cat. CLS3450) and PC membranes with 0.4 µm pores (Merck Millipore, Darmstadt, Germany, Millicell, cat. PIHP03050) from commercial inserts were cut out of their inserts, in the following referred to as PET0.4 and PC0.4, respectively. Samples (*N* ≥ 3) were taken from different membranes that were cast on multiple days. According to standard protocol, the membranes were prewetted overnight in 100% ethanol, after which they were stabilised by placing them in an Amicon cell (Merck Millipore, Darmstadt, Germany, Amicon cell 8003). The PET membranes were used without prewetting with ethanol since they performed better this way. A transmembrane pressure (TMP) of 1 bar for M0, M1 and PET0.4 samples, and 0.3 bar for M2, M3 and PC0.4 samples was then applied. Afterwards, the pressure was lowered, and the amount of permeated water was measured at different TMP, i.e., 0.3, 0.5 and 1 bar for M0, M1 and PET0.4 samples, and 0.1, 0.2 and 0.3 bar for M2, M3 and PC0.4 samples. The flux of the latter membranes was difficult to measure at higher TMP due to the high flow through these membranes. Measurements were performed for at least 15 min for each TMP. Water permeance was then determined as the slope of the best linear fit of the flux versus TMP for each sample.

### 2.7. Statistical Analysis

Statistical analysis was done with a One-way-ANOVA with a Bonferroni post-hoc test in Graphpad Prism5 software (Graphpad, San Diego, CA, USA) with *p* < 0.05. Capped lines with asterisks mark significant differences in graphs.

## 3. Results and Discussion

### 3.1. Membrane Fabrication

For the membrane fabrication, the basic polymer dope contained PTMC, chloroform, PETA, Irgacure 2959, PEO and a non-solvent. We first optimised the polymer dope composition and EIPS conditions for the fabrication of porous PTMC membranes. 

#### 3.1.1. PEO and Non-Solvent Type

Based on earlier studies on PTMC-based scaffolds [31,37], we added PEO as pore former and stabiliser in the PTMC dope, which led to porous membranes (results not shown). Moreover, PEO improved membrane handling by making the membranes less sticky. Therefore, PEO was included in all polymer dopes at a PTMC to PEO ratio of 1:0.1 *w*/*w* following Papenburg et al. [31]. 

**Figure 1:**
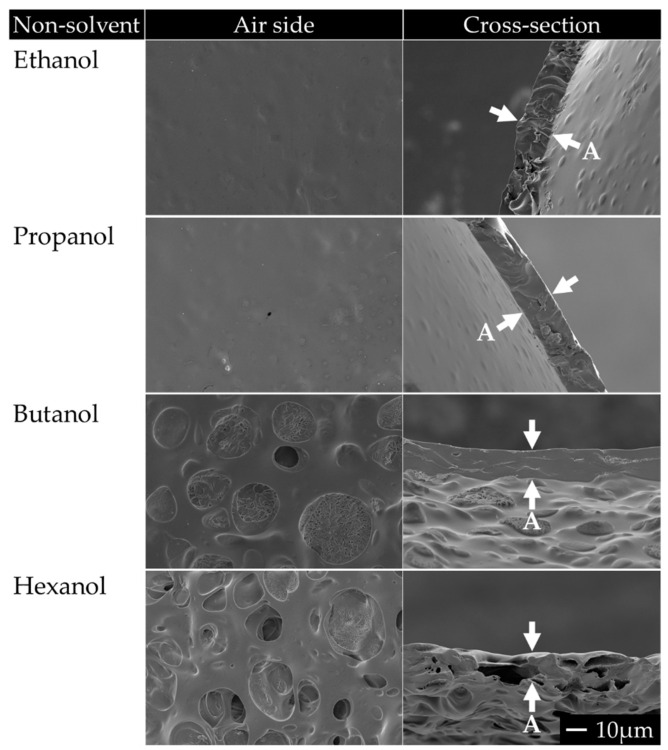
SEM images of membranes made from PTMC and different alcohols in the polymer dope as non-solvents. The ratio of PTMC to non-solvent was 1:1 (*w*/*w*). Magnification: 1000×. Scale bar applies to all images. The cross-section of the membranes is shown between arrows. An ‘A’ adjacent to an arrow marks the air side of the membrane. Images are representative of observations of multiple membranes.

We also investigated the effect of adding various non-solvents in the PTMC polymer dope for the production of porous PTMC membranes. For this, we prepared polymer dopes with PTMC (MW = 1300 kg/mol) and different alcohols as non-solvents, i.e., ethanol, propanol, butanol or hexanol. Figure 1 shows SEM images of the air side (i.e., the side facing the ambient air where most of the chloroform evaporates) and of the cross-section of the produced membranes. The application of small, volatile alcohols, i.e., ethanol and propanol, as non-solvents mainly resulted in non-porous membranes, except for a few superficial ‘pores’ in the case of propanol. The application of butanol resulted in membranes with shallow pore-like structures on the air side only, while the other parts of the membranes were non-porous. The application of hexanol, however, resulted in porous PTMC membranes, at the surface and across the membrane (Figure 1). This is likely because the less volatile hexanol remained present in the entire cast polymer layer, allowing proper coalescence, which resulted in pore formation throughout the entire membrane. 

These results are in agreement with other studies which highlighted the need for low non-solvent evaporation for achieving porous membranes via EIPS [44,45]. Pervin et al. showed that when the non-solvent evaporation is increased at high temperature, EIPS resulted in membranes with only surface pores [45]. Non-solvent evaporation and subsequent diffusion to the air side likely also occurred during the fabrication of our membranes, mainly with butanol, propanol and ethanol. This was stimulated on top of the PTMC membranes by the airflow applied across the surface. Despite evaporation, the concentration of butanol at the air side was high enough to result in limited pore formation. Residual amounts of the more volatile propanol and ethanol were probably too small for EIPS to occur. Based on these results, hexanol was used as the non-solvent in further membrane fabrication.

#### 3.1.2. Ambient Humidity and Substrate Temperature

We also performed preliminary studies on the effect of humidity and temperature of the casting plates on the morphology of the PTMC membranes. Membranes with 3 wt% 1300 kg/mol PTMC and 3 wt% hexanol were cast on silicon wafers at 50% humidity or 60% humidity, the latter created by a humidifier. Afterwards, the cast membranes and silicon wafers were placed on glass plates of 19–20 °C or −25 °C. 

We found that membrane porosity increased both at relatively high ambient humidity of 60% during EIPS and by placement on the cold glass plates, as well as both combined (results not shown). These conditions likely hindered chloroform evaporation, thus slowing down EIPS and allowing more time for hexanol to coalesce, resulting in larger pore size and higher porosity of the membranes, consistent with other studies [44,45]. Furthermore, evaporation of the solvent and possibly non-solvent leads to a decrease in the temperature of the air side, also known as the evaporative cooling effect [45]. This stimulates water deposition from the ambient air onto the membrane surface. This water deposition can already lead to pores at the air side [45] and is the main principle of pore formation in (water-)vapour-induced phase separation (VIPS) [46,47,48]. Higher humidity of the ambient air could promote this effect, as was shown by Pervin et al. [45]. Thus, high ambient humidity, as well as placement on cold glass plates during casting and EIPS, were used in further membrane fabrication.

#### 3.1.3. PTMC Molecular Weight

Subsequently, we investigated the effect of the MW of PTMC on the fabrication of porous membranes. Polymer dopes were prepared consisting of the basic components mentioned previously, with 3 wt% hexanol as non-solvent and PTMC with a MW of either 1300 kg/mol or 600 kg/mol (Table 1). We then cast membranes on wafers and performed EIPS in a humid environment on cold plates as described above. The polymer dopes with PTMC of 1300 kg/mol were quite viscous, and the PTMC was incompletely dissolved, resulting in heterogeneous membranes (Figure S1). In contrast, the PTMC with a MW of 600 kg/mol could be dissolved well, and the produced membranes were homogeneous. Besides, these membranes had larger pores and higher porosity. The coalescence of hexanol was likely better due to the lower viscosity of the polymer dope and thus higher molecular mobility, similar to the results reported by Zhao et al. [44].

Pervin et al. investigated the formation of porous poly(methyl methacrylate) (PMMA) membranes via EIPS and applied PMMA with a MW of 15 kg/mol and 120 kg/mol and mixtures of both. They reported that application of 15 kg/mol PMMA resulted in non-porous membranes [45]. In contrast, they found pore formation when the PMMA mixtures were used. Moreover, higher amounts of 120 kg/mol PMMA led to pore formation deeper in the membranes. They suggested that the 15 kg/mol PMMA was too soluble, and thus EIPS was only initiated in the presence of the 120 kg/mol PMMA and when the concentration of 120 kg/mol PMMA was sufficiently high.

They also reported that polymer dopes with more than 40 wt% PMMA had a high viscosity, which prevented homogeneous mixing leading to heterogeneous polymer dopes [45]. This is consistent with our findings with the high MW PTMC (1300 kg/mol), where the resulting PTMC membranes were heterogeneous due to the high polymer dope viscosity. The PTMC concentration in our polymer solutions follows Papenburg et al. [31]. Lower PTMC concentrations, as used in studies with relatively thick PTMC scaffolds [49,50], may decrease heterogeneity in the case of the 1300 kg/mol PTMC, but will likely also result in very thin and fragile membranes. Moreover, Pervin et al. showed that a very low polymer concentration could limit the occurrence of EIPS [45]. Conversely, increasing the polymer concentration in the case of the 600 kg/mol PTMC would likely result in denser membranes and similar problems as with 1300 kg/mol PTMC due to a too high viscosity of the polymer solution. Therefore, the PTMC concentration in the polymer solutions was kept at 3 wt%.

Based on these results, the MW of the polymer needs careful consideration for obtaining porous and homogeneous membranes via EIPS. Application of low MW polymer (having high solubility and low viscosity) can lead to non-porous membranes, while application of high MW polymer (having low solubility and high viscosity) can lead to non-homogeneous porous membranes. Based on our results, the PTMC with a MW of 600 kg/mol is the most suitable for obtaining porous membranes. Therefore, it was used in further experiments.

### 3.2. The Effect of the Non-Solvent Amount

Here, we prepared polymer dopes containing PTMC of 600 kg/mol and different amounts of hexanol to tailor the membrane properties. Membranes are referred to as M0–M3 based on the ratio of PTMC to hexanol in the polymer dope, i.e., 1:0 to 1:3 (*w*/*w*), respectively (see also in Section 2.3 and Table 2).

#### 3.2.1. The Polymer Network in the Membranes

Since optimal membrane crosslinking is desired for achieving stable porous PTMC membranes, we assessed the polymer network integrity of the developed membranes. Table 2 presents the gel content of the membranes, which expresses the percentage of the membranes that was crosslinked. Increasing amounts of hexanol in the polymer dope resulted in membranes with lower gel content, i.e., an average gel content of 95.9% to 52.9% for M0–M3. The lower gel content could be due to the partial dissolution of the crosslinking agents in the hexanol, which limited their availability and thus decreased crosslinking efficiency and gel content. Higher hexanol content in the polymer dope probably increased this effect. However, due to the relatively low amounts of non-solvent, the gel content after EIPS and crosslinking was still high enough to provide membranes with good integrity. In fact, the M1–M3 membranes could be handled well when placed in cell culture liquids, i.e., PBS and cell culture media (results not shown). Besides, these membranes could be wetted easily, but did not swell significantly in these liquids and were not adhesive. 

^1^H-NMR analysis of the sol fraction of membranes indicates that uncrosslinked PTMC and PEO were present in the membranes (Figure S2). However, there was no detectable hexanol or chloroform (Figure S3), nor Irgacure 2959 and PETA (Figure S4), suggesting that they were removed during washing or, in the case of PETA, crosslinked in the membranes. PTMC and PEO were likely both crosslinked through hydrogen abstraction [32,51]. Together with multifunctional acrylic radicals from the PETA [32], this resulted in a network of PETA, PEO and PTMC. Uncrosslinked PTMC and PEO were likely entangled in the polymer network due to their high MW and could not be removed completely by washing with ethanol and water.

Similar findings of entrapped PEO in PTMC scaffolds were reported by Papenburg et al. [31] who used 266 kg/mol PTMC and PEO of 6000 kg/mol to fabricate porous PTMC scaffolds by LIPS. After washing the scaffolds in MilliQ water, there was still PEO present. They proposed that during phase separation, the viscosity of the PTMC-rich phase increased to a point where some of the PEO could be entrapped in the PTMC phase. The viscosity of our polymer dopes was likely higher since our PTMC had a substantially higher MW, while the concentration and MW of PEO were similar. Moreover, our membranes were crosslinked for at least 52.9%. Therefore, uncrosslinked PTMC and PEO were likely only removed when the membranes were swollen in chloroform.

#### 3.2.2. Mechanical Properties of Membranes

Table 2 also features the E-moduli of M0–M3 membranes which were in the range of 9–14 MPa. There was no significant difference between M0–M3 membranes. This further confirmed that the decreasing gel content did not influence the stability or mechanical properties of the membranes. The E-moduli of the PTMC membranes were significantly lower than those of the previously mentioned PET and PC membranes (2–3 GPa [11] and 2–2.4 GPa [12], respectively), but similar to those of some PDMS membranes (4 kPa to several MPa [22]). 

Bat et al. [32] made films from high MW PTMC that were crosslinked similarly to our M0–M3 membranes. Those films with similar gel content and E-moduli (approximately 10 MPa) to our membranes had a stress at break up to more than 30 MPa, an elongation at break up to more than 600% and a permanent deformation of 2% or less. These results indicated good toughness and the capability of being used for cyclic stretch.

As established before, the mechanical properties of PTMC structures are well preserved in a wet state [37,38]. The E-modulus could decrease in a wet state [38], which would be beneficial for our application. Moreover, the M0–M3 membranes have low swelling, as shown by the experiments in cell culture liquids, and thus also a low water uptake. Therefore, it is unlikely that the mechanical properties of our membranes would be negatively affected by wetting.

#### 3.2.3. Porosity, Morphology and Water Transport

**Figure 2:**
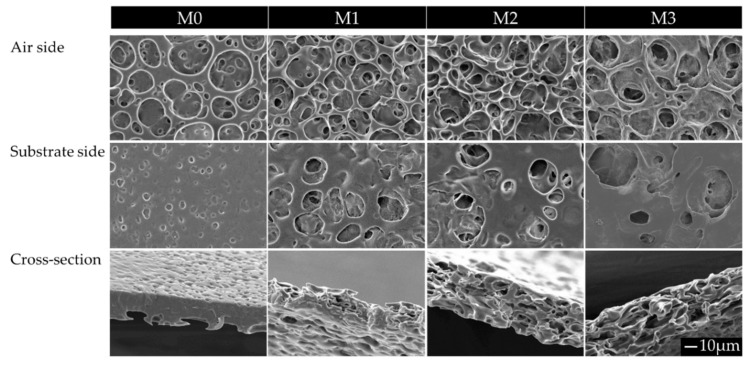
SEM images of membranes made from PTMC with a MW of 600 kg/mol with different hexanol content in the polymer dope. Magnification: 1000×. Scale bar applies to all images. Images are representative of observations of multiple membranes (*N* ≥ 7).

**Figure 3:**
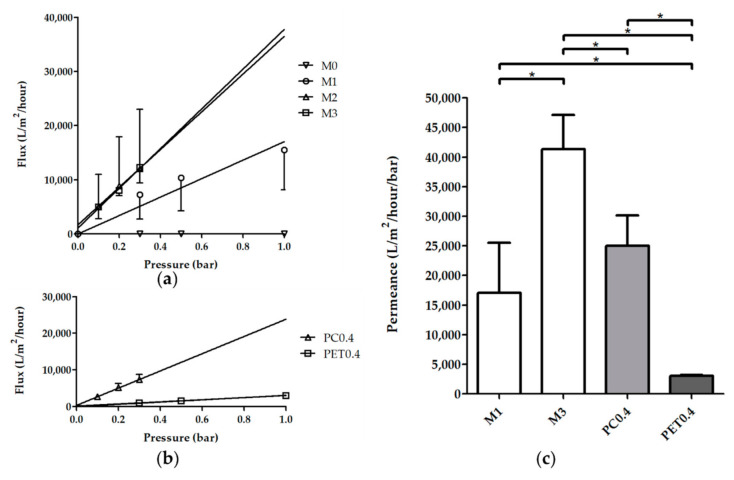
Effect of the hexanol amount on the water transport across M0–M3 PTMC membranes. PC and PET membranes with 0.4 µm pores taken from commercial inserts (PC0.4 and PET0.4, respectively) were used as a reference. (**a**,**b**) Water flux of membranes measured at different TMP after pre-compaction. M2, M3 and PC0.4 samples were measured at lower pressures since their flux was too high to collect at higher pressures. Shown are average fluxes ± SD (*N* ≥ 3), lines represent the best linear fit. (**a**) Demonstrates M0–M3, (**b**) shows PC0.4 and PET0.4 samples. (**c**) The permeance of the different membranes was calculated by determining the best linear fit of the water flux data for each sample and calculating the average. M0 membranes are not shown since they were not permeable to water. Depicted are averages ± SD (*N* ≥ 3). Capped lines with asterisks above the graph show significant differences (*p* < 0.05). M2 membranes were not included in the statistical analysis due to the high variation of their water fluxes.

Membranes in both Transwell^®^ inserts and various OoCs require sufficient porosity to allow nutrient transport to the cultured cells as well as cell-cell signaling by secreted mediators when culturing cells on either side of the membrane. Therefore, we investigated how to tailor the overall porosity, pore morphology and pore connectivity across the membranes (Table 2 and Figure 2 and Figure 3).

##### Overall Porosity and Thickness

The M1–M3 membranes were porous, whereas the M0 membranes were non-porous (Table 2). The hexanol concentration in the polymer dope increased overall porosity from 21.1% in M1 membranes to 33.4% in M2 membranes and 41.5% in M3 membranes. However, although there were differences between the M1 membranes and the M2 and M3 membranes, all porosities were relatively low. That could explain why the E-moduli did not change between the M1–M3 membranes. The M2 and M3 membranes were also thicker than the M0 membranes (Table 2) due to their porosity. 

##### Membrane Morphology

Figure 2 and Table 2 show SEM images and pore sizes, respectively, of the air side, the substrate side and the cross-sections of M0–M3 membranes. M0 membranes were mainly non-porous, with only a few pores on the air side and in the cross-section of some samples (Figure 2). The pores did not seem to interconnect due to the low pore density as was apparent by the cross-section of the M0 membranes. Moreover, the substrate sides of the samples completely lacked pores, thus preventing any interconnection between the air and substrate side of the M0 membranes. M1–M3 membranes had a spongy structure with seemingly interconnected pores and higher overall porosity and thickness (Figure 2), consistent with the results of the overall porosity and thickness (Table 2). The porosity was higher at the air side than the substrate side (Figure 2). The diffusion of chloroform towards the air side, caused by its evaporation from the surface, likely extended the duration of EIPS at the air side. This probably stimulated the coalescence of hexanol, resulting in higher porosity at the air side than the substrate side. The pore sizes on the air side of M1 and M2 membranes (5.2 ± 3.1 and 5.3 ± 2.1 µm, respectively) were smaller than those on the air side of M3 membranes, i.e., 7.9 ± 4.1 µm (Table 2). Thus, increasing the hexanol amount in the dope increased the surface pore size. 

Pores on the air side of M1 and M2 membranes were smaller compared to those on their respective substrate sides and in their cross-sections. Perhaps the deposition of water droplets on the air sides of the membranes limited the coalescence of hexanol compared to the substrate sides and cross-sections, since hexanol and water do not mix well. The additional hexanol in M3 membranes may have been enough to avoid the effect of the water. Moreover, phase separation likely occurred earlier in M3 membranes than in M1 and M2 membranes because of the higher non-solvent concentration, as was suggested by Zhao et al. [44]. Thus, EIPS started in M3 membranes when there was less deposition of the water droplets and thus less interference with coalescence compared to EIPS in the M1 and M2 membranes. 

Pore shape also seemed to become less circular as the hexanol concentration was increased (Figure 2). While M1 membranes and to a lesser extent M2 membranes featured mostly circular pores, the shape of pores in M3 membranes was more irregular. The latter may have been caused by better coalescence of multiple larger hexanol droplets on the air side of M3 membranes compared to that in M1 and M2 membranes.

Consistent with the increasing pore size seen at the surface of M1 to M3 membranes, Zhao et al. showed that increasing the non-solvent concentration in the polymer dope led to larger pores and lower pore density [44]. As mentioned, they suggested that a higher non-solvent amount initiated EIPS faster. EIPS, therefore, occurred at a higher solvent concentration and thus at a lower viscosity, which can stimulate coalescence of the non-solvent. Thus, at higher non-solvent content, there are more favorable conditions for the coalescence of the non-solvent as well as more non-solvent present, which both contribute to the increased pore size.

In general, M0–M3 membranes had a relatively smooth substrate side (Figure 2), likely due to the contact with the smooth silicon wafers. In contrast, the air side of the membranes had higher roughness and contained crater-like structures. These structures resembled the honeycomb structures also found in membranes fabricated with VIPS and were likely caused by water deposition on the membranes [45,46,47,48] as a result of the evaporative cooling effect mentioned earlier [45]. The humid and relatively cold casting conditions and the presence of hydrophilic PEO likely stimulated deposition of condensed water droplets on the M0–M3 membranes. This may also explain why the membranes featured in Figure 1 had relatively smooth air sides. Since those membranes were cast at 19–20 °C and 50% humidity, whereas the M0–M3 membranes were cast at −25 °C (at the start of chloroform evaporation) and 60% humidity, the deposition of water droplets was probably limited in the former case. 

The M1–M3 membranes were opaque (Figure S5), but became more transparent upon wetting. The water in the pores may help light travel through the pores. Standard cell culture inserts with PC membranes with 0.4 µm pores (PC0.4) had a comparable level of transparency. In any case, application of M1–M3 membranes in in vitro organ models, e.g., OoCs, is possible, where the cells can often be viewed from multiple sides under the microscope. Moreover, a live cell tracker could be used to track the cells, if necessary.

The opaqueness of M1–M3 membranes increased when the concentration of hexanol in the polymer dope was increased (Figure S5). M1 and M3 membranes were macroscopically homogeneous, while M2 membranes were heterogeneous having both transparent and opaque areas across the entire surface area. Zhao et al., who determined the phase diagrams for their silicon rubber membranes made by EIPS [44], proposed that EIPS occurred via nucleation and growth at low and high non-solvent concentration, or via spinodal decomposition at certain non-solvent concentrations in between the former. Possibly, phase separation in the case of the M1 and M3 membranes occurred through nucleation and growth, whereas in the case of M2 membranes, EIPS took place via spinodal decomposition. The unstable phase separation in the latter case is probably the reason for the heterogeneous membrane morphology of M2 membranes compared to the M1 and M3 membranes.

##### Water Transport Across the Membranes

Figure 3 shows the water flux (Figure 3a,b) and the estimated water permeance (Figure 3c) of M0–M3 membranes compared to PC0.4 and PET membranes with 0.4 µm pores (PET0.4). As expected, the non-porous M0 membranes had no water flux. However, M1–M3 membranes had high water permeance in the microfiltration range, i.e., 17,000–41,000 L/m^2^/h/bar, which confirmed that the pores were well interconnected. 

The average fluxes of M2 and M3 membranes were similar. These membranes already showed a water flux at a TMP of 0 bar due to the hydrostatic pressure of the water. Furthermore, water flux increased linearly with TMP in M1–M3 (Figure 3), indicating no membrane compaction and good stability of the porous networks. 

The water fluxes of the M2 membranes had a larger variation than those of M1 and M3 membranes indicating a larger variation in the pore interconnectivity of the M2 membranes, consistent with the observations made earlier about membrane heterogeneity. Due to this high variation, M2 membrane water transport data were excluded from the statistical analysis of the estimation of the water permeance of the different membranes. For comparison, the PET0.4 membranes had much lower water permeance than M1 and M3 membranes, whereas the PC0.4 membranes had a permeance similar to the M1 membranes. Besides, the permeance of the M3 membranes was higher than that of the M1 and PC0.4 membranes.

### 3.3. EIPS and Photo-Crosslinking for Tailoring of Membrane Properties

In this study, we combined for the first time EIPS and photo-crosslinking to produce form-stable, porous and permeable PTMC membranes. Our method could be an interesting option to fabricate porous polymer structures from amorphous polymers. The membranes had similar porosity, pore size and thickness as LIPS-based PTMC membranes [31]. In general, the membrane pore sizes were comparable to those of PDMS membranes used in some OoCs [1,2,10]. LIPS, although well-known, simple and used for many applications, is less favorable for the purposes mentioned above, since polymer crosslinking via gamma-irradiation can be difficult [31] and photo-crosslinking is likely not straightforward. 

Here, we prepared mechanically stable porous membranes with very good porosity, even at relatively low gel contents of 52.9% to 79.2%, due to the entanglement of the PTMC and PEO. However, if lower MW polymers would be used, the obtained gel contents may be too low for proper membrane integrity since there would be less entanglement of the polymers. In those cases, it would be relevant to raise the crosslinking efficiency, which can be achieved by adding more crosslinking agents to the polymer dope [32].

Moreover, EIPS and photo-crosslinking could be used with other amorphous or semi-crystalline polymers and copolymers (e.g., TMC and ε-caprolactone (ε-CL) [52] or poly(dl-lactic acid) (PDLLA) and ε-CL [53]) with good cell adhesive properties to develop porous membranes.

In this study, we were able to make membranes with high water permeance (in the microfiltration range, i.e., 17,000–41,000 L/m^2^/h/bar) similar to that of commercial PC inserts. Thus, our membranes are likely well-suited for application in cell culture systems and OoCs, allowing nutrient delivery and paracrine signaling of cells on opposite sides of the membranes. Permeance was raised by increasing the non-solvent concentration in the polymer dope. This effect was likely due to better pore interconnectivity since raising the non-solvent concentration increased overall porosity and pore size on the air side of membranes. Moreover, the permeances were high for membranes with a rather low porosity of 21.1–41.5%. The relatively low porosities and pore sizes of our membranes may stimulate cells to grow in proper confluent cell layers and thus create better barriers compared to membranes or scaffolds with much higher porosities and pore sizes [32,43,44], which may discourage cell-cell contact. 

The easy wetting and high permeances of the M1–M3 membranes suggest that these membranes are quite hydrophilic, which could be partially due to the presence of hydrophilic PEO at the surface. However, since the membrane surface also contains hydrophobic PTMC, the hydrophilicity is likely moderate. This is advantageous for cell culturing since moderate hydrophilicity has been shown to promote cell adhesion, due to good adsorption of cell adhesive proteins [1].

The PTMC membranes developed here have E-moduli of approximately 10 MPa, which are much lower than the E-moduli of commonly used materials such as PET and PC and similar to those of several tissues [15,17,18]. This is an important step forward in the development of tissue-mimicking supports for cell culturing. In the future, the E-moduli could be further decreased by the use of lower PETA concentrations [29] or a diacrylate instead of a triacrylate, such as PETA [39]. Besides, EIPS and photo-crosslinking could be used with copolymers of e.g., TMC and ε-CL, since networks of these copolymers have lower E-moduli than networks of either polymer alone [52]. 

## 4. Conclusions

Organ models often use culture membranes with properties that do not match those of the represented tissue. Methods to create membranes with suitable pore sizes from flexible but low cell adhesive PDMS are often elaborate, while more easily applicable methods often require stiff and/or low cell adhesive materials. This study shows, for the first time, the development of porous, form-stable PTMC membranes by combining EIPS and photo-crosslinking. Important parameters for tailoring the membrane fabrication, such as polymer dope additives, humidity, and the type and amount of non-solvent were investigated. We could adapt the membrane pore size on the air side and the pore connectivity by increasing the non-solvent content in the polymer dope. The resulting M1 and M3 membranes both had high water permeance similar or higher than that of commercial membranes, good mechanical properties and suitable pore sizes for application in in vitro organ models. These characteristics make both the M1 and M3 membranes promising as cell culture supports for use in such models. Moreover, the obtained understanding of EIPS and its established compatibility with photo-crosslinking could be used to prepare membranes from other amorphous or semi-crystalline polymers for biomedical applications.

## Figures and Tables

**Table 1:** Components used in polymer dopes.

Function of Component	Trivial Name/Abbreviation	Component (IUPAC Name)	MW	Wt% of Total Polymer Dope
			**kg/mol**	
**Polymer**	PTMC	Poly(trimethylene carbonate)	1300	3
			600	
**Pore stabiliser**	PEO	Poly(ethylene oxide)	5000	0.3
**Crosslinking agents:**				
**Crosslinking aid**	Pentaerythritol triacrylate (PETA)	[2-(hydroxymethyl)-3-prop-2-enoyloxy-2-(prop-2-enoyloxymethyl)propyl] prop-2-enoate	0.298	0.2
**Photoinitiator**	Irgacure 2959	2-hydroxy-1-[4-(2-hydroxyethoxy)phenyl]-2-methylpropan-1-one	0.224	0.01
**Solvent**	Chloroform	Trichloromethane	0.119	96.5–87.5
**Non-solvent**	Ethanol	Ethanol	0.046	3
	Propanol	Propan-1-ol	0.060	3
	Butanol	Butan-1-ol	0.074	3
	Hexanol	Hexan-1-ol	0.102	0–9

**Table 2:** PTMC membrane properties as a result of different hexanol amounts in the polymer dopes. Polymer dopes were made using chloroform as a solvent. Dopes always contained PTMC with a MW of 600 kg/mol, PEO, PETA and Irgacure 2959. The remaining part of the polymer dope either consisted solely of chloroform or an amount of hexanol, complemented by chloroform (see 2nd and 3rd rows). The table shows different membrane properties and the effect of hexanol content in the polymer dope on these properties. Data were analysed by One-way ANOVA. Significant differences (*p* < 0.05) with other membranes are marked by (M0–M3) underneath the data referring to those membranes. Significant differences in pore size between parts of a specific membrane are marked by (A), (S) or (Cr), indicating air side, substrate side or cross-section, respectively. Pore size analysis was based on ≥7 images per condition.

Membrane		M0	M1	M2	M3
**Hexanol** **(wt% of polymer dope)**		0	3	6	9
**Chloroform** **(wt% of polymer dope)**		96.5	93.5	90.5	87.5
**Gel content (%)** **(*N* ≥ 3)**		95.9 ± 2.0 _(M1,M2,M3)_	79.2 ± 7.3 _(M0,M2,M3)_	60.4 ± 4.8 _(M0,M1)_	52.9 ± 5.6 _(M0,M1)_
**E-modulus (MPa)** **(*N* ≥ 2)**		10.4 ± 1.6	14.0 ± 5.3	14.3 ± 1.5	9.4 ± 2.4
**Overall porosity (%)** **(*N* ≥ 4)**		- _(M1,M2,M3)_	21.1 ± 5.4 _(M0,M2,M3)_	33.4 ± 6.8 _(M0,M1)_	41.5 ± 8.5 _(M0,M1)_
**Thickness (µm)** **(*N* ≥ 6)**		18.2 ± 4.1 _(M2,M3)_	20.3 ± 3.4 _(M2,M3)_	31.4 ± 4.6 _(M0,M1)_	36.6 ± 7.7 _(M0,M1)_
**Average pore size (µm)** **(*N* ≥ 7)**	Air side	1.9 ± 0.5 _(M1,M2,M3)_	5.2 ± 3.1 _(M0,M3) (S,Cr)_	5.3 ± 2.1 _(M0,M3) (S,Cr)_	7.9 ± 4.1 _(M0,M1,M2)_
	Substrate side	- _(M1,M2,M3)_	7.2 ± 4.0 _(M0) (A)_	7.8 ± 3.3 _(M0) (A)_	6.9 ± 3.2 _(M0)_
	Cross-section	1.4 ± 1.0 _(M1,M2,M3)_	8.0 ± 4.3 _(M0) (A)_	7.4 ± 3.4 _(M0) (A)_	7.6 ± 4.3 _(M0)_

## Supplementary Materials

The following are available online at https://www.mdpi.com/2077-0375/10/11/330/s1, Figure S1: Images of macroscopic morphology of dry PTMC membranes made from PTMC with a MW of 1300 kg/mol or 600 kg/mol, Figure S2: ^1^H-NMR spectra of the sol fraction of M0–M3 membranes, pure PTMC and pure PEO, Figure S3: ^1^H-NMR spectra of the sol fraction of M0–M3 membranes, pure chloroform-D and pure hexanol, Figure S4: ^1^H-NMR spectra of the sol fraction of M0–M3 membranes, pure PETA and pure Irgacure 2959, Figure S5: Images of macroscopic morphology of dry M0–M3 PTMC membranes made from PTMC with a MW of 600 kg/mol and different hexanol concentrations in the polymer dope.
