# Supplementary material for: Development of Porous and Flexible PTMC Membranes for In Vitro Organ Models Fabricated by Evaporation-Induced Phase Separation
Source: Membranes (Basel). 2020 Nov 5;10(11):330. doi: 10.3390/membranes10110330 (PMC7694515; doi:10.3390/membranes10110330)
Supplement: Supplementary file 1 [file membranes-10-00330-s001.zip › membranes-976155-supplementary.docx]

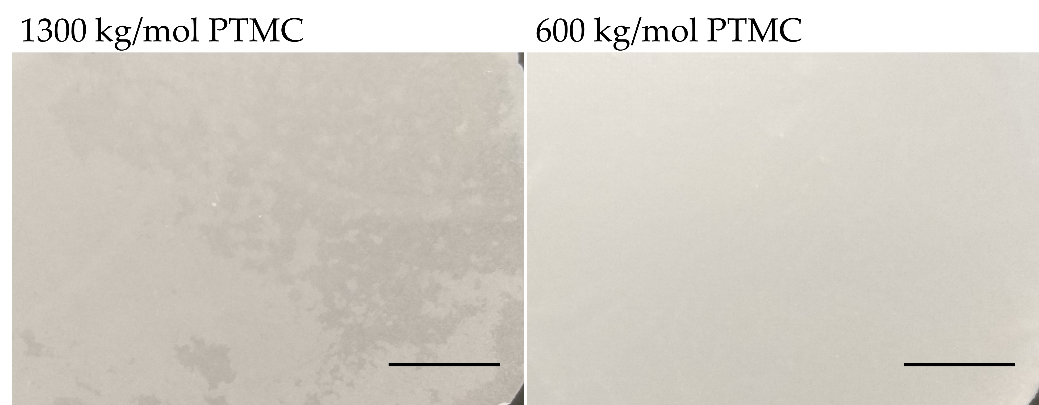


**Figure S1.** Images of macroscopic morphology of dry PTMC membranes made from PTMC with a MW of 1300 kg/mol or 600 kg/mol. The ratio of PTMC to hexanol in the polymer dope was 1:1 (*w/w*). Scale bar: 1 cm. Membranes made from 1300 kg/mol PTMC were heterogeneous, containing both transparent (dark) and opaque (light) areas indicating differences in porosity, while those made from 600 kg/mol PTMC were homogeneous. Images are representative of multiple observations of different membranes.


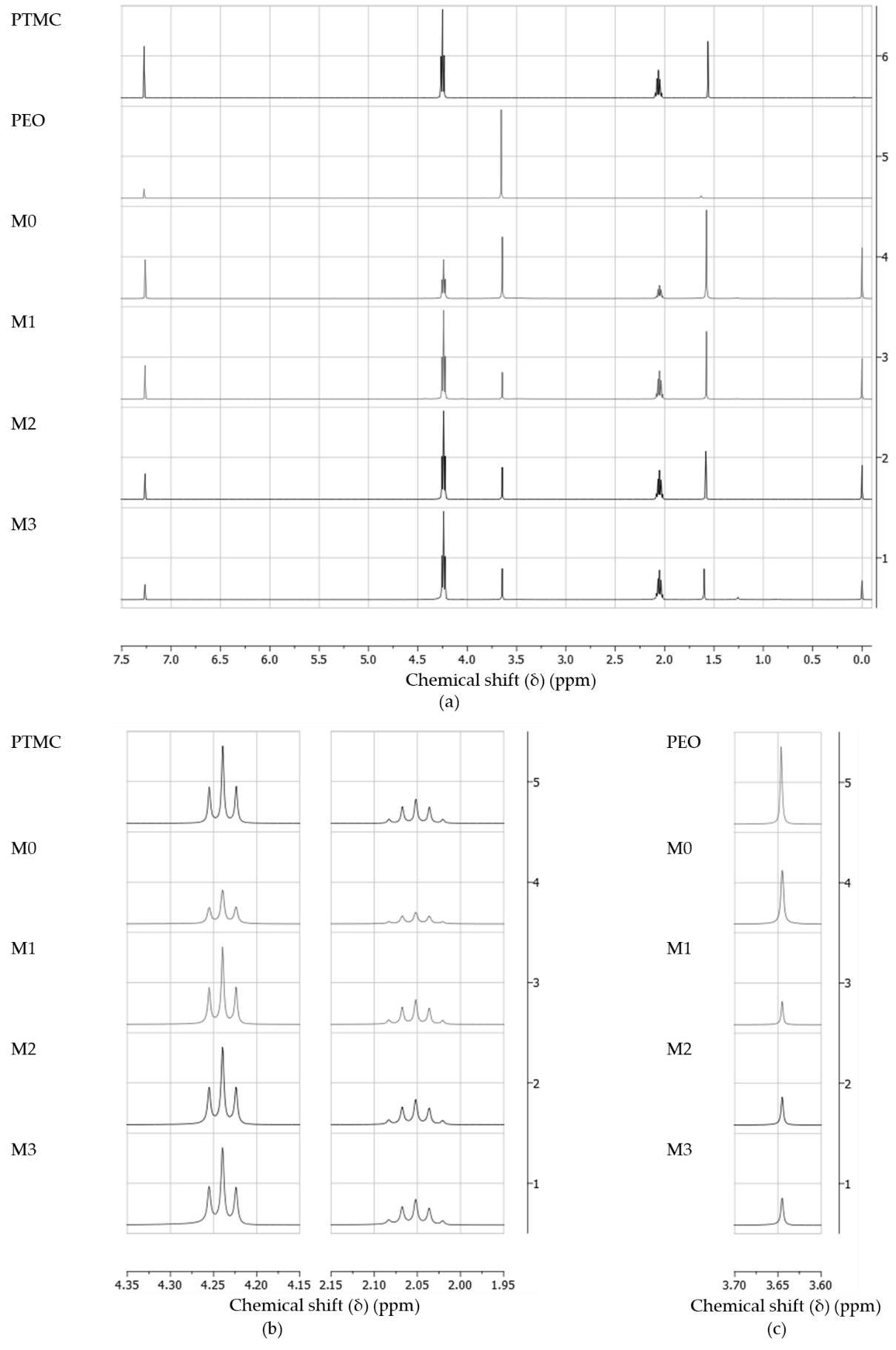


**Figure S2.** ^1^H-NMR spectra of the sol fraction of M0–M3 membranes, pure PTMC and pure PEO. **(a)** Full spectra of the sol fraction of M0–M3 membranes, PTMC and PEO. **(b)** Partial spectra of the sol fraction of M0–M3 membranes and PTMC showing characteristic peaks for PTMC. The presence of the triplet around 4.24 ppm and the quintet around 2.06 ppm in the sol fraction of M0–M3 membranes confirms that uncrosslinked PTMC was present in the membranes. **(c)** Partial spectra of the sol fraction of M0–M3 membranes and PEO showing a characteristic peak for PEO. The presence of the singlet around 3.64 ppm in the sol fraction of M0–M3 membranes confirms that uncrosslinked PEO was present in the membranes. Spectra are representative of multiple samples (*N* ≥ 5 for all membranes and *N* = 3 for the pure components).


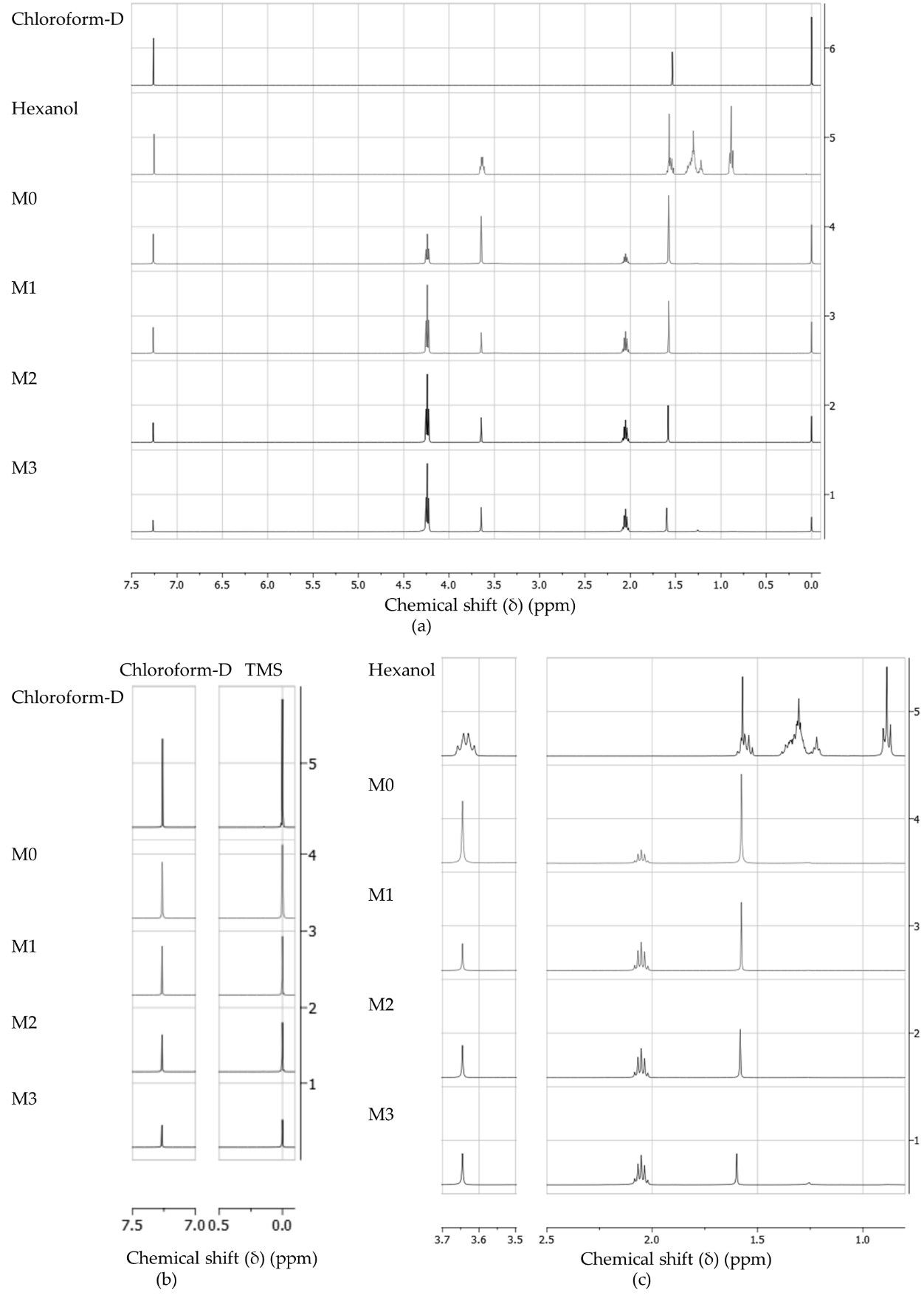


**Figure S3.** ^1^H-NMR spectra of the sol fraction of M0–M3 membranes, pure chloroform-D and pure hexanol. **(a)** Full spectra of the sol fraction of M0–M3, chloroform-D and hexanol. **(b)** Partial spectra of the sol fraction of M0–M3 membranes and chloroform-D showing chloroform and tetramethylsilane (TMS), a reference in the chloroform-D with a peak at 0.0 ppm, in the samples. The ratio between chloroform and TMS did not change, suggesting that M0–M3 membranes contained no chloroform. **(c)** Partial spectra of the sol fraction of M0–M3 membranes and hexanol showing characteristic peaks for hexanol. The absence of a quartet around 3.64 ppm and several multiplets between 1.6 and 0.8 ppm in the sol fraction of M0–M3 membranes confirms that hexanol was not present in the membranes. Spectra are representative of multiple samples (*N* ≥ 5 for all membranes and *N* = 3 for the pure components).


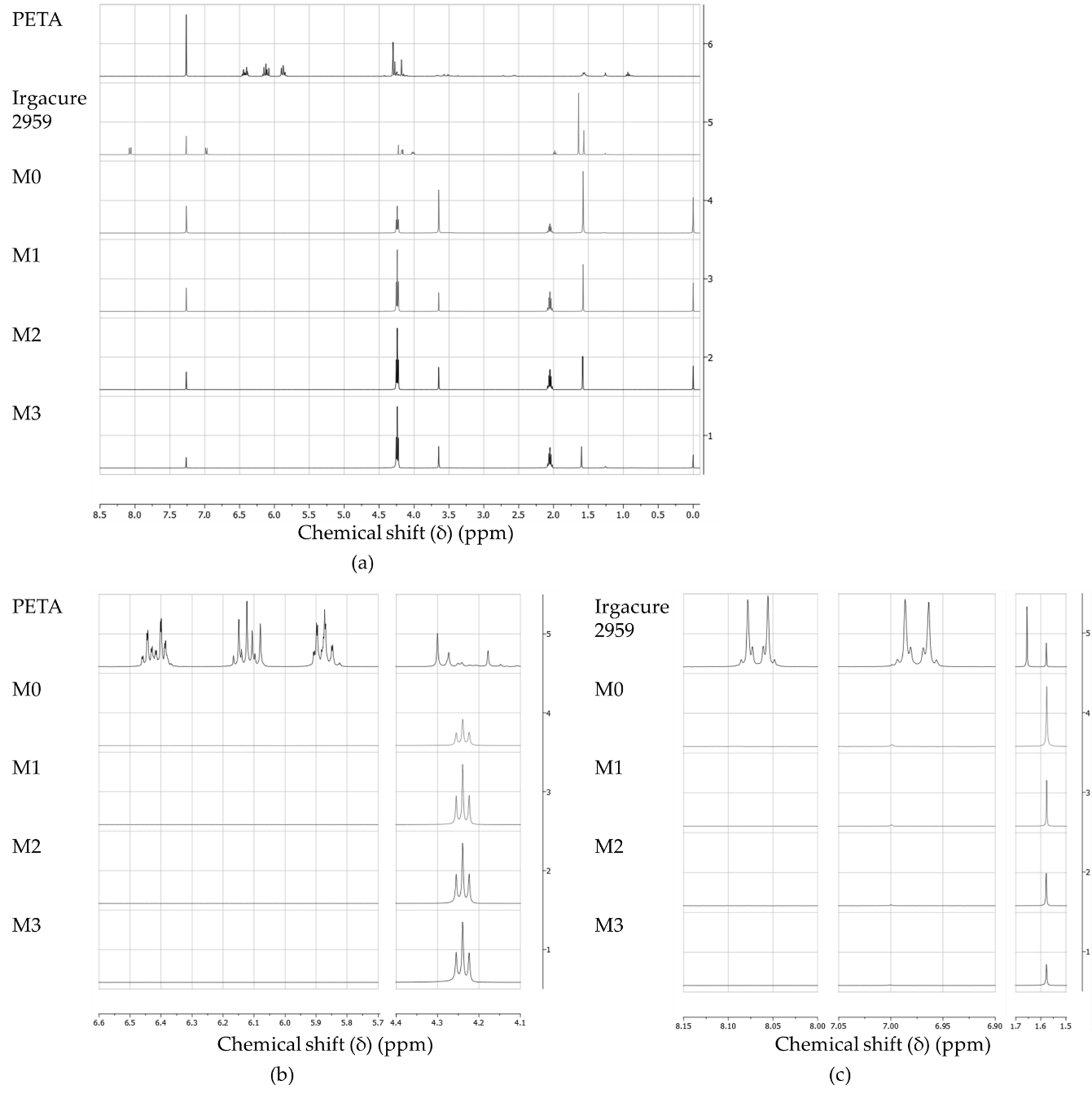


**Figure S4.** ^1^H-NMR spectra of the sol fraction of M0–M3 membranes, pure PETA and pure Irgacure 2959. **(a)** Full spectra of the sol fraction of M0–M3 membranes, PETA and Irgacure 2959. **(b)** Partial spectra of the sol fraction of M0–M3 membranes and PETA showing characteristic peaks for PETA. The absence of the multiplets between 6.5 and 5.8 ppm and the peaks between 4.4 and 4.1 ppm in the sol fraction of M0–M3 membranes confirms that uncrosslinked PETA was not present in the membranes. **(c)** Partial spectra of the sol fraction of M0–M3 membranes and Irgacure 2959 showing characteristic peaks for Irgacure. The absence of the double triplets between both 8.10–8.00 and 7.00–6.95 ppm, as well as the peak at 1.65 ppm in the sol fraction of M0–M3 membranes confirms that uncrosslinked Irgacure was absent in the membranes. Spectra are representative of multiple samples (*N* ≥ 5 for all membranes and *N* = 3 for the pure components).


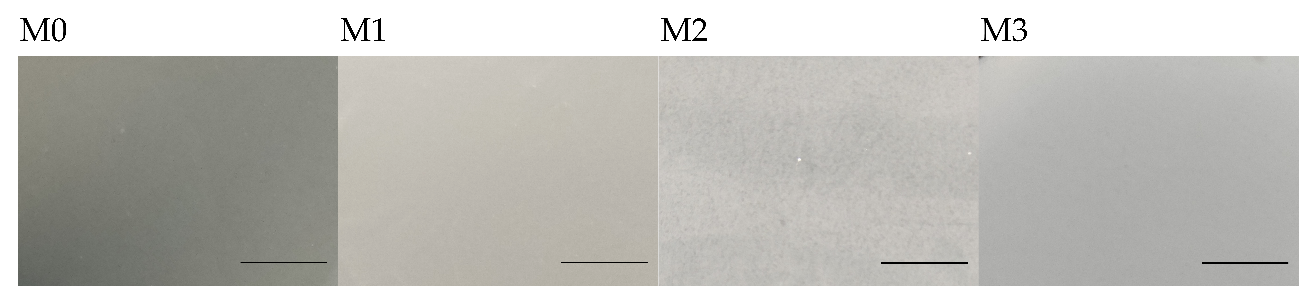


**Figure S5.** Images of macroscopic morphology of dry M0–M3 PTMC membranes made from PTMC with a MW of 600 kg/mol and different hexanol concentrations in the polymer dope. Scale bar: 1 cm. M0 membranes were transparent, while M1 and M3 membranes were opaque. M0, M1 and M3 membranes were all homogeneous. M2 membranes, however, had a heterogeneous morphology, having both transparent and opaque areas throughout the membranes. Images are representative of multiple observations of different membranes.
